# Does Electroacupuncture Treatment Reduce Pain and Change Quantitative Sensory Testing Responses in Patients with Chronic Nonspecific Low Back Pain? A Randomized Controlled Clinical Trial

**DOI:** 10.1155/2018/8586746

**Published:** 2018-10-08

**Authors:** Paula M. S. Leite, Andreza R. C. Mendonça, Leonardo Y. S. Maciel, Maurício L. Poderoso-Neto, Carla C. A. Araujo, Hilda C. J. Góis, Jérsica H. S. Souza, Josimari M. DeSantana

**Affiliations:** ^1^Graduate Program in Health Sciences, Federal University of Sergipe, 49.060-100, Brazil; ^2^Department of Physical Therapy, Federal University of Sergipe, 49.100-000, Brazil; ^3^Graduate Program in Physiological Sciences, Federal University of Sergipe, 49.060-100, Brazil

## Abstract

Chronic nonspecific low back pain is common and one of the most disabling conditions in the world. There is moderate evidence that chronic low back pain patients present altered functional connectivity in areas related to pain processing. Quantitative sensory testing is a way of clinical measure of these alterations. Although there is not enough evidence, there are some reports that electroacupuncture is supposedly more effective in relieving pain than acupuncture because the addition of electric current could optimize the effects of traditional technique. Thus, the objective of this randomized clinical trial was to verify if electroacupuncture treatment reduces pain and changes quantitative sensory testing responses in patients with chronic nonspecific low back pain. Patients were evaluated before and after 10 sessions regarding pain (11-point numerical rating pain scale) and quantitative sensory testing (pressure pain threshold, temporal summation, and conditioned pain modulation). There were 1 treatment group (electroacupuncture (EA)) and three different control groups (CTR 1, CTR 2, and CTR 3). A total of 69 patients participated in the study. No significant differences were found in pain intensity or quantitative sensory testing responses when comparing electroacupuncture group to the three control groups. There was a significant reduction in both resting and movement pain intensity in groups EA, CTR 1, and CTR3. Although ten sessions of electroacupuncture have diminished pain intensity in both resting and movement, it could not change significantly quantitative sensory testing and diminish central sensitization in patients with chronic nonspecific low back pain. The implications of this study involve the fact that, maybe, in chronic nonspecific low back pain, electroacupuncture should be associated with other treatments that target central sensitization.

## 1. Introduction

Chronic nonspecific low back pain is common and one of the most disabling conditions in the world [1, 2]. According to a systematic review, there is moderate evidence that chronic low back pain patients present brain structural changes in both gray and white matter and also altered functional connectivity in areas related to pain processing [3]. These neuroplastic modifications may be clinically assessed thru quantitative sensory testing, as there is preliminary to moderate evidence demonstrating relationship between clinical pain measures and those structural and functional connectivity alterations in chronic musculoskeletal patients [4].

Some available and commonly used quantitative sensory tests in musculoskeletal disorders are the investigation of mechanical detection threshold, heat detection threshold, vibration detection threshold, and pressure pain threshold [5]. They also allow assessing primary and secondary hypoesthesia or hyperalgesia, which are mechanisms involved with pain transmission thru dorsal horn neurons [6]. Also, quantitative sensory testing is a way of measuring central hypersensitivity or impaired endogenous pain modulation mechanisms, like temporal summation of pain and conditioned pain modulation, respectively.

While traditional acupuncture dates back at least 2,000 years ago, electroacupuncture is a relatively recent technique, since it has been started to be used in the last 50 years [7]. One of the advantages of electroacupuncture in the clinical practice or research is its ability to objectively and quantifiably define stimulus frequency and intensity [7]. Although there is insufficient evidence, there are some reports that electroacupuncture is supposed to be more effective for pain relief than manual acupuncture, since the addition of electric current could optimize the effects of the traditional technique [8, 9].

Microinjection of the beta-endorphin antagonist into the periaqueductal gray matter (PAG) decreased electroacupuncture-induced analgesia, suggesting that this neurotransmitter is involved on its mechanism of action [10]. In addition, other neurotransmitters are involved such as cholecystokinin 8 (CCK 8), at 100 Hz; endorphin, at 2 Hz; enkephalin and dynorphin at both 2 and 100 Hz; endomorphin, at 2 Hz, and substance P, at 10 Hz [6, 11]. In a study using magnetic resonance imaging, the difference in activation of areas between low and high frequency of electroacupuncture was minimal [7].

Although there is a lot of published systematic reviews, meta-analyses, guidelines, and increasing research funding on low back pain, patients' self-reported disability have not improved in the last years [12]. Regarding electroacupuncture, there is no previous article that has analyzed this intervention in an isolated way for treating chronic nonspecific low back pain or without other chronic diseases being included in the treatment group.

In addition, although acupuncture is an age-old therapy and has shown to have good results in the clinical practice, there are still some gaps in the clinical trials that make it unclear whether electroacupuncture differs from acupuncture or whether it is just a variation of the technique, or even if there is some advantage by adding an electric stimulus to the already inserted needles.

## 2. Methods

### 2.1. Study Design

This is a double-blinded and placebo controlled randomized clinical trial. Distribution was made with sealed opaque envelopes containing numbers 1 to 4, corresponding to the number of groups, performed in a blocked proportion of 1:1. For assuring the blinding process, there were two types of investigators in this study: investigator 1, responsible for evaluating patients and measuring all variables, before and after treatment; and investigator 2, responsible for applying the treatment during all sessions. Neither the patient nor investigator 1 knew in which group (real or control) subjects were allocated. Patients were treated at the Ambulatory of Laboratory for Research in Neurosciences, located at Federal University of Sergipe.

This study follows Standards for Reporting Interventions in Clinical Trials of Acupuncture (STRICTA) recommendations.

### 2.2. Eligibility Criteria

Patients were included if they had (1) low back pain diagnostic made by an orthopedics physician; (2) pain on the lumbar region for at least three months; (3) never been submitted to acupuncture or electroacupuncture treatment previously. Exclusion criteria were (1) doing physiotherapy or other treatment for low back pain; (2) being pregnant or postpartum women who had given birth in the past three months; (3) having deformities or important amputations on lower limbs; (4) having low back pain due to infection, tumor, osteoporosis, rheumatoid arthritis, vertebrae fracture, or radiculopathy; (5) having nervous or cutaneous tissue injury affecting lumbar region; (6) having active infectious processes; (7) having surgery or invasive exams on the spine on the past three months; (8) having inability to understand instructions or consent to the study; (9) having psychiatric or cognitive impairments; (10) having neurological (i.e., stroke, Parkinson's, Alzheimer's, cerebral tumor, dementia, and multiple sclerosis), pulmonary (i.e., chronic obstructive pulmonary disease (COPD)), or cardiac (i.e., arrhythmia,* angina pectoris*, congestive heart failure, and decompensated hypertension) disease; (11) having heart pacemaker; (16) having auditory, visual, or communication disturbance.

For sample size calculation, it was considered pain intensity measured by the 11-point numerical rating pain scale with previous data from our pilot study: standard deviation = 2, difference to be detected = 2, significance level = 5%, and test power = 80%. A minimum of 17 subjects was needed for each group, considering a total of 68 patients.

### 2.3. Ethical Aspects

This study was approved by the Committee of Ethics for Research in Humans of the Federal University of Sergipe (CAAE 32193214.4.0000.5546, report number 716.611) and also registered on the Brazilian Registry of Clinical Trials (report number: RBR-3w2p32). It complies determinations of 466/12 Resolution from the Brazilian National Health Council. All subjects included assigned an informed consent before to be included in the study.

### 2.4. Study Groups

Patients were randomly allocated into one of the four study groups and Traditional Chinese medicine style was used in all groups. Randomization was done with opaque sealed envelopes in the proportion 1:1. There were 1 treatment group (EA) and 3 control groups, considering electroacupuncture as a focus in this study. Patients in the treatment group received electroacupuncture (needle + electric current).

A 10 Hz frequency was chosen because it decreases substance P; and at 100 Hz, dynorphin is released [6, 13]. Both 10 and 100 Hz diminishes hyperalgesia thru *μ* and *δ* opioid receptors [13]. During the 30-minute stimulation, frequency was alternated between high and low every 5 seconds. Maximum sensory intensity (as soon as motor threshold was reached, then current intensity was diminished) was used.

In Control Group 1, electrical stimulus lasted only 45 seconds, but needles were still kept inserted during 30 minute-period. Patients in Control Group 2 received only had a needle inserted in, without electrical stimulus or device (needle alone). In Control Group 3, needles were placed in the same other group's acupoints; however, they were withdrawn immediately after puncture. Patients in this group could not see that needles were removed.

Acupoints were used in all groups. These points were chosen because they are commonly used to treat low back pain in clinical practice. As this study has a mechanistic approach, all patients received puncture in the same points. Sterile acupuncture needles, 25 x 30 mm sized (Suzhou Huanqiu Acupuncture Medical Appliance Co. Ltd.®), were inserted bilaterally during 30 minutes in 4 acupuncture points related to low back pain: (1) B22, located 1,5 cm laterally to L1 vertebrae; (2) B26, located 1,5 cm laterally to L5 vertebrae; (3) B50, located 3 cm laterally to T12 vertebrae; (4) B53, located 3 cm laterally to S2 vertebrae. In the CTR 3 group, needles were inserted in those same acupuncture points; however, they were immediately removed after puncture.

This method has been previously tested by our group comparing different types of placebos and real acupuncture. No difference was found between groups when needling sensation was compared [14]. In the EA group, a device (Sikuro DS 100c - Sikuro Sistemas e Equipamentos Eletrônicos Ltda, Rio de Janeiro, RJ) that generates the electrical current was coupled to needles. Three experienced, trained, and licensed acupuncturists for more than eight years delivered treatment and a total of 8 needles (depth of 10 mm) were used per patient in each session [14]. Patients had similar body mass index and they were not obese (BMI<30 kg/cm^2^), that is why in needle could pass thru the skin or fat. Treatment was delivered three times a week (Mondays, Wednesdays, and Fridays) in a total of ten sessions. No additional components of treatment (moxibustion, cupping, and herbs) were used. Patients were informed that different types of acupuncture were being compared in the study. All patients allocated in placebo groups received the same treatment as the active groups when all sessions and data collection have ended.

### 2.5. Measurement Methods

#### 2.5.1. Pain

Pain intensity was measured by using the 11-point numerical rating pain scale that ranges from 0 to 10, with 0 indicating “no pain” and 10 “worst pain imaginable”. Patients verbally classified their pain at rest (standing position) and during flexion-extension movement of lumbar spine (patient was in a standing position and then was instructed to try to touch his fingers on his toes).

Brazilian version of McGill Pain Questionnaire [15] was used to quantify and to characterize pain. Patients were instructed to choose one (or none) word that mostly described perceived pain in each twenty categories. Then, the number of words chosen (NWC) and pain rating index (PRI) were calculated according to patient's answers and were used to compare results between treatment groups.

#### 2.5.2. Quantitative Sensory Testing

Four quantitative sensory test measures were used to characterize patients' pain processing: (1) tactile detection threshold (TDT), (2) pressure pain threshold (PPT), (3) temporal summation of pain (TS), and (4) conditioned pain modulation (CPM).

TDT was measured with a kit of twenty von Frey monofilaments (North Coast®, Gilroy, California, USA). Monofilament was positioned perpendicularly to patients' skin and then a light pressure, sufficient to bend monofilament in a “U” format, was done [16]. Patients, with eyes closed, were instructed to inform when they felt the monofilament touch. If no answer was given, investigator applied other filament with bigger diameter. Filaments were applied in crescent order. Calibration was previously done in the same way test was applied, using a precision balance (CQA®, Paulínia, São Paulo, Brazil). Values registered in grams were converted to milinewton (mN).

Test was applied in two points bilaterally: (A) referent to the local of pain (primary hypoesthesia), located at the midpoint of paravertebral muscle belly, at the level of the third lumbar vertebrae and (B) distant point from pain area (secondary hypoesthesia), at the muscle belly of tibialis anterior muscle, at the level of tibialis anterior tuberosity [17]. Three measures were done and then media was registered.

In these same points and after TDT measures, PPT was evaluated with a pressure algometer with a probe area of 1 cm^2^ (EMG System®, São José dos Campos, SP, Brazil). With algometer positioned perpendicularly to patients' tissue, a crescent pressure was done and patient was instructed to inform when pressure clearly became painful. An interval of one minute was done between each one of the three measures.

TS was measured by applying a constant pressure of 4 kg/cm^2^ in a point 7,5 cm from wrist line. Pain intensity was asked verbally thru the 11-point numerical rating pain scale during the 1st, 10th, 20th, and 30th second of stimulation.

To evaluate CPM, firstly, PPT was measured in the right forearm, 7,5 cm from wrist line. An ischemic compression of 270 mmHg was made in the contralateral arm with an sphygmomanometer (Mikatos®, Embu, SP, Brazil) positioned 3 cm proximally to the cubital fossa, then patient opened and closed left hand 10 times. Pain intensity was asked and when it was equal or more than 4, PPT was measured in the right arm, during the ischemic compression. Five minutes after this procedure, PPT was again measured, now without compression.

In all groups, patients were instructed not to take analgesics, anti-inflammatories, or opioids.

### 2.6. Statistical Analysis

Initially, data collected were transported to a spreadsheet in Excel for Windows 2010, where the descriptive statistics were performed, with measures of position (mean, median, minimum, and maximum) and dispersion (standard error of the mean). Subsequently, comparisons made between and within groups were made in the program Statistical Package for the Social Sciences (SPSS) version 16.0.

All data were tested for normality by Shapiro-Wilk test. Categoric data were analyzed by qui-square test. In intragroup analysis, all variables were nonparametric and were analyzed by Wilcoxon test. When comparing groups, data that followed a normal distribution (weight, height, BMI, age, and pain time) were analyzed by ANOVA. All the other variables followed a nonnormal distribution and were analyzed using the Kruskal-Wallis test followed the Tukey post hoc test.

In all comparisons, it was considered statistically significant difference when the p value of the analysis was less than 0.05. The values were expressed as mean and standard error of the mean.

## 3. Results

**Figure 1:**
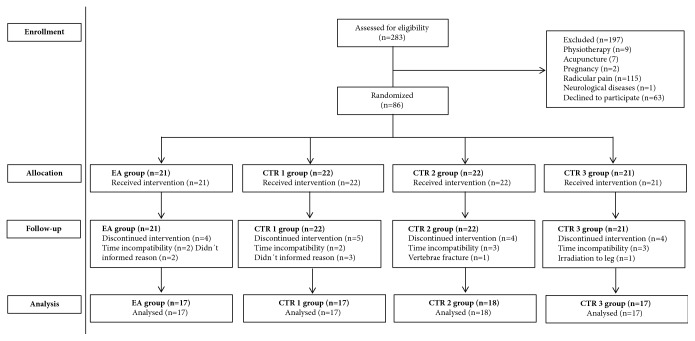
Study flow diagram.

A total of 283 individuals were assessed for eligibility. 197 subjects were excluded because they did not met inclusion criteria or declined to participate.  Figure 1 summarizes reasons of exclusion and discontinued intervention. A total of 69 patients participated in the study (EA: 17; CTR 1: 17; CTR 2: 18; CTR 3: 17).

No significant differences were found between groups regarding demographic characteristics (Table 1).

Also, no significant differences (p≥0.05) were found in pain intensity or quantitative sensory testing responses when comparing electroacupuncture group to the three control groups (Table 2). There was a significant reduction in both resting and movement pain intensity in groups EA, CTR 1, and CTR 3.

## 4. Discussion

In this study, electroacupuncture treatment could not change quantitative sensory testing responses in patients with chronic nonspecific low back pain. This treatment reduced pain intensity in both rest and movement; however it also occurred in CTR 1 and, curiously, in CTR3, where needle was immediately removed after puncture.

According to the Revised Standards for Reporting Interventions in Clinical Trials of Acupuncture (STRICTA), an extension of the CONSORT statement [18], this minimal stimulus present in CTR 3 may elicit some neurophysiological, localized immune and/or circulatory changes that may reduce pain, but the specific mechanisms are still unknown [18]. Notwithstanding, recent meta-analysis [19] concluded that sham or placebo acupuncture were more efficacious for chronic nonspecific low back pain relief than routine care or waiting list. Studies that assessed chronic pain patients included in this meta-analysis were similar to the present study regarding the type of acupuncture, duration, and number of sessions.

Despite this, pain reduction in group CTR 3 may have happened not only because of the possible effect that minimal penetration can elicit, but also due to the placebo effect. Patient's beliefs that they might be receiving an active treatment can influence responsiveness of treatment such that, according to an overview of systematic reviews [20], some results from countries where acupuncture is widely used cannot be expanded to places in which acupuncture is just an alternative practice.

Sham and placebo procedures should be similar to real acupuncture and the ideal was that they still were physiologically inert. Meeting this both criteria is not easy for acupuncture studies [19]. In our study, we used puncture + immediate removal technique in CTR 3. Maciel et al. [14] compared the placebo effect between different nonpenetrating acupuncture devices, needling + immediate needles withdrawn, and real acupuncture in healthy 321 healthy volunteers that were randomly divided into 14 groups that received puncture in the abdominal point stomach ST [21] or the lumbar point bladder (Bl) 52 for stimulation. No significant differences were found regarding general perception of acupuncture, discomfort at the moment of puncture, location of the feeling of puncture, and intensity of discomfort caused by the puncture. Also, no significant differences were found between real and placebo groups when subjects were asked if he or she believed had received a real or placebo procedure.

Conversely, an overview of systematic reviews and randomized clinical trials about noninvasive treatments for chronic low back pain found that acupuncture is associated with lower pain intensity than sham and no acupuncture, with moderate magnitude of effect and low strength of evidence for pain outcome [20]. In this same review, compared with medicines (nonsteroidal anti-inflammatory drugs: NSAIDs, muscle relaxants, and analgesics), acupuncture was associated with better pain relief, but with small magnitude of effect and low strength of evidence. So, although there is no strong evidence, maybe it is better to use acupuncture than medicines, also taking into account the fact that the harm effects from acupuncture are minimal. Mechanisms by which electroacupuncture and manual-acupuncture insertion reduced pain intensity in the present study involve opioids release, proinflammatory cytokines reduction, and decrease of phosphorylation in n-methyl-d-aspartate receptor [13].

Other systematic review [22] also attributed magnitude of effects to noninvasive interventions in chronic low back pain. It was based on mean between-group differences. Slight/small magnitude was defined as a difference of 0.5–1.0 points on a 0- to 10-point numerical rating scale or the equivalent. Differences between 1.0 and 2.0 points were classified as moderate and >2 points were categorized as large/substantial. In our study, no statistic differences were found between groups; however, resting pain intensity in electroacupuncture group was at least 1 point lower than CTR 1 and CTR 2, so magnitude could be classified as moderate, in accordance with the study cited previously. The same could not happen in CTR 3, where difference was 0.20 (small magnitude). For movement pain, magnitude was moderate for CTR 1 and small for CTR 2 and CTR 3.

In the present study, the addition of electrical current to needles was not superior to the other groups. A study by Napadow et al. [7] with a functional magnetic resonance imaging compared the central effects of EA in different frequencies with traditional manual acupuncture. Three active acupuncture stimuli produced more regions of positive and negative hemodynamic signal than the control group. However, EA produced a greater increase in signal than manual acupuncture [7]. Despite this, this was a study conducted in healthy subjects so it is not known if the presence of pain could alter this result.

In the present study, it was expected to verify a reduction in both primary and secondary hyperalgesia, since a modulated frequency associating low stimulation frequencies, which reduce secondary hyperalgesia, while higher frequencies reduce primary hyperalgesia, was used [6].

Nonetheless, even using modulated frequency in the present study, maybe ten sessions of electroacupuncture were not sufficient to reduce central sensitization. Studies have suggested that pain related to hypoesthesia is probably due to central plasticity, as a consequence of nociceptive activity [21] and MDT is a way of measuring somatosensory system [23]. In the present study, mechanical detection threshold did not change significantly. EA also could not diminish PPT primary and secondary hyperalgesia or increase endogenous pain inhibition in conditioned pain modulation. Yet, central sensitization is a complex phenomenon which involves biological, psychological and social patterns [24], so treatment for it should involve a variety of approaches.

Interestingly, temporal summation of pain, a measure of central facilitation [25], had its magnitude partly diminished only in groups electrically stimulated, even in CTR 1, where device was turned on for only 45 seconds. TENS, another treatment that uses peripheral electrical current can also do this in chronic pain patients. It is known that electroacupuncture has some effects in central nervous system, but brain mechanisms of electroacupuncture pain reduction need to be more investigated [13].

However, in general, electroacupuncture was not able to significantly alter quantitative sensory testing responses and diminish central sensitization in patients with chronic nonspecific low back pain in this study. Notably, there are a few noninvasive treatments with promising results for reducing central sensitization symptoms in musculoskeletal disorders, such as TENS [26] and exercise [27]. Both of them activate inhibitory descending pathway, then reducing impairments on endogenous pain inhibition systems.

In the last years, most recent guidelines have been recommending self-management, active, physical, and psychological therapies, instead of surgical and pharmacological treatments. Acupuncture is suggested as second-line or adjunctive treatment option for some guidelines, but others do not recommend it at all [28, 29]. However, ponderation has to be done because all interventions (acupuncture, medication, exercise, and manual therapy) should receive equal classification criteria and in some guidelines it was not present [30]. American College of Physicians recommended that patients initially should receive nonpharmacological treatments and acupuncture is one of these, with moderate-quality evidence and strong recommendation, regarding the classification developed by the GRADE (Grading of Recommendations Assessment, Development and Evaluation workgroup) [31].

To our knowledge, this is the first randomized clinical trial that investigated the effect of electroacupuncture in central pathways of patients with chronic nonspecific low back pain. Hence, more efforts are needed to improve the internal and external validity of systematic reviews and randomized clinical trials about acupuncture and electroacupuncture in low back pain [32].

Limitations of this study involve a lack of control group without receiving treatment, for example, waiting list. Also, a comparison with some usual care group should be interesting. Besides that, acupuncture and electroacupuncture have an individual approach. In clinical practice, acupuncture points are chosen according to individual/individuality characteristics. However, in trials with a mechanistic approach, as this study, standardization and methodological rigor are needed [18].

## 5. Conclusions

Although ten sessions of electroacupuncture have diminished pain intensity in both resting and movement, it could not change significantly quantitative sensory testing and diminish central sensitization in patients with chronic nonspecific low back pain. The implications of this study involve the fact that, maybe, in chronic nonspecific low back pain, electroacupuncture should be associated with other treatments that target central sensitization.

## Figures and Tables

**Table 1:** Sample characterization. BMI: Body Mass Index. CTR: control group. Data expressed as mean and standard error of the mean. p>0.05 in all comparisons. Kruskal-Wallis test for noncategoric variables and qui-square test for categoric.

**Demographic characteristics**	**Electroacupuncture**	**CTR 1**	**CTR 2**	**CTR 3**
**Age (years)**	42.35±3.35	41.82±3.34	48.72±3.61	52.58±3.65
**Weight (kg)**	71.73±2.22	72.80±2.35	76.56±2.64	79.08±3.10
**Height (m) **	1.67±0.02	1.68±0.02	1.66±0.02	1.68±0.03
**BMI (kg/m** ^**2**^ **)**	25.62±0.79	25.82±0.78	27.75±0.99	27.97±0.76
**Sex (**%**)**				
**Female**	10 (58.8%)	9 (52.9%)	10 (55.5%)	9 (52.9%)
**Male**	7 (41.2%)	8 (47.1%)	8 (44.5%)	8 (47.1%)
**Occupation (**%**)**				
**Long sitting**	6 (35.2%)	6 (35.2%)	4 (22.2%)	5 (29.4%)
**Long standing**	9 (52.9%)	9 (52.9%)	11 (61,1%)	8 (47.0%)
**Retiree**	2 (11.9%)	2 (11.9%)	3 (16.7%)	4 (23.6%)
**Physical activity (**%**)**				
**Yes**	10 (58.8%)	8 (47.1%)	10 (55.5%)	10 (58.8%)
**No**	7 (41.20%)	9 (52.9%)	8 (44.5%)	7 (41.2%)
**Smoking (**%**)**				
**Yes**	0 (0%)	1 (5.9%)	0 (0%)	0 (0%)
**No**	17 (100%)	16 (94.1%)	18 (100%)	17 (100%)
**Alcohol consumption (**%**)**				
**Yes**	4 (23.5%)	6 (35.3%)	3 (16.7%)	4 (23.6%)
**No**	13 (76.5%)	11 (64.7%)	15 (83.3%)	13 (76.4%)

**Table 2:** Pain intensity and quantitative sensory testing in patients with chronic nonspecific low back pain before and after treatment. NWC: number of words chosen. PRI: pain rating index. TDT: tactile detection threshold. TAM: tibialis anterior muscle. PPT: pressure pain threshold. CTR: control group. Data expressed as mean and standard error of the mean. *∗*p<0.05. *∗∗*p<0.01. Wilcoxon matched pairs test.

	**Electroacupuncture**		**CTR 1 (needle + device off)**		**CTR 2 (needle alone)**		**CTR 3 (withdrawn needles)**	
	**Before**	**After**	**Before**	**After**	**Before**	**After**	**Before**	**After**
**Resting pain (0 – 10)**	3.61 ± 0.64	1.33 ± 0.46*∗*	4.40 ± 0.56	2.69 ± 0.42*∗∗*	3.16 ± 0.47	2.06 ± 0.49	3.47 ± 0.45	1.53 ± 0,45l*∗∗*
**Moviment pain (0 – 10)**	4.33 ± 0.55	1.55 ± 0.51*∗∗*	5.25 ± 0.48	2.69 ± 0.41*∗∗*	3.11 ± 0.74	2.00 ± 0.47	3.41 ± 0.75	1.93 ± 0,57*∗*
**NWC McGill (0 – 20)**	18.24 ± 0.55	16.75 ± 1.16	18.11 ± 0.60	15.75 ± 1.35	18.00 ± 0.53	16.15 ± 0.60*∗∗*	17.25 ± 0.67	11.84 ± 1.76*∗*
**PRI McGill (0 – 78)**	34.81 ± 2.16	30.37 ± 2.96	34.33 ± 1.85	27.62 ± 2.37	37.40 ± 2.29	27.65 ± 1.67*∗∗*	31.95 ± 2.22	24.41 ± 3.92*∗*
**TDT right lumbar (mN)**	0.147 ± 0.024	0.095 ± 0.025	0.174 ± 0.026	0.099 ± 0.007	0.080 ± 0.007	0.087 ± 0.014	0.153 ± 0.053	0.077 ± 0.006
**TDT left lumbar (mN)**	0.128 ± 0.022	0.090 ± 0.024	0.135 ± 0.020	0.098 ± 0.013	0.148 ± 0.049	0.137 ± 0.050	0.116 ± 0.200	0.105 ± 0.020
**TDT right TAM (mN)**	0.233 ± 0.071	0.148 ± 0.051	0.254 ± 0.062	0.131 ± 0.020	0.104 ± 0.015	0.087 ± 0.014	0.253 ± 0.073	0.176 ± 0.052
**TDT left TAM (mN)**	0.179 ± 0.056	0.104 ± 0.019	0.226 ± 0.051	0.175 ± 0.034	0.109 ± 0.025	0.137 ± 0.030	0.284 ± 0.074	0.207 ± 0.058
**PPT right lumbar (kgf)**	3.52 ± 0.52	3.48 ± 0.46	3.86 ± 0.49	3.38 ± 0.34	3.18 ± 0.35	2.92 ± 0.35	0.317 ± 0.41	3.54 ± 0.46
**PPT left lumbar (kgf)**	3.36 ± 0.44	3.42 ± 0.43	4.03 ± 0.48	3.58 ± 0.36	3.02 ± 0.38	2.92 ± 0.35	3.00 ± 0.43	3.83 ± 0.52
**PPT right TAM (kgf)**	3.16 ± 0.32	3.57 ± 0.33	3.93 ± 0.45	3.66 ± 0.32	3.07 ± 0.39	2.88 ± 0.37	3.13 ± 0.48	3.66 ± 0.56
**PPT left TAM (kgf)**	3.18 ± 0.35	3.55 ± 0.35	3.97 ± 0.51	3.51 ± 0.30	3.02 ± 0.37	2.82 ± 0.38	3.16 ± 0.46	3.46 ± 0.44
**Temporal summation 1” (0 – 10)**	4.12 ± 0.73	2.37 ± 0.34*∗∗*	4.16 ± 0.60	2.87 ± 0.46*∗*	3.60 ± 0.63	2.93 ± 0.30	3.58 ± 0.54	2.35 ± 0.50
**Temporal summation 10” (0 – 10)**	5.31 ± 0.69	3.75 ± 0.36*∗*	6.00 ± 0.59	4.25 ± 0.49*∗*	4.75 ± 0.65	4.00 ± 0.43	5.23 ± 0.53	3.78 ± 0.54
**Temporal summation 20” (0 – 10)**	6.18 ± 0.69	4.75 ± 0.41	6.61 ± 0.54	5.12 ± 0.55*∗*	5.51 ± 0.61	4.33 ± 0.46	6.35 ± 0.54	4.21 ± 0.62
**Temporal summation 30” (0 – 10)**	6.68 ± 0.71	6.06 ± 0.54	7.33 ± 0.66	6.18 ± 0.56	6.04 ± 0.61	5.20 ± 0.50	7.05 ± 0.48	5.00 ± 0.66
**Conditioned pain modulation pre (kgf)**	3.21 ± 0.52	3.57 ± 0.45	3.26 ± 0.40	3.19 ± 0.38	2.84 ± 0.34	2.90 ± 0.28	3.52 ± 0.44	3.00 ± 0.39
**Conditioned pain modulation during (kgf)**	3.50 ± 0.54	3.71 ± 0.30	3.50 ± 0.42	3.06 ± 0.36	2.78 ± 0.24	2.99 ± 0.29	3.53 ± 0.38	3.07 ± 0.38
**Conditioned pain modulation after (kgf)**	3.14 ± 0.35	3.83 ± 0.44	3.34 ± 0.33	3.39 ± 0.40	2.86 ± 0.33	2.67 ± 0.25	3.59 ± 0.50	2.74 ± 0.23

## Data Availability

The data used to support the findings of this study are available from the corresponding author upon request.
